# Unlocking the mysteries of n-oxPTH: implications for CKD patients

**DOI:** 10.3389/fendo.2024.1455783

**Published:** 2025-01-03

**Authors:** Lina Zhang, Huixia Cao

**Affiliations:** Department of Nephrology, Henan Key Laboratory of Kidney Disease and Immunology, Henan Provincial People’s Hospital, Zhengzhou, Henan, China

**Keywords:** oxidized, parathyroid hormone, chronic kidney disease, prognosis, immunoassay

## Abstract

Parathyroid hormone (PTH) is a pivotal hormone that regulates serum calcium and phosphate and is closely associated with higher risk of cardiovascular disease and mortality in patients with chronic kidney disease (CKD). PTH can undergo oxidation at methionine 8 and methionine 18 of the molecule. This oxidation process leads to a lower binding affinity to the PTH receptor due to molecular refolding, particularly for PTH oxidized at methionine 8. Although, the oxidation of PTH has been reported for several decades, it is only recently that a method has been developed to detect non-oxidized PTH (n-oxPTH) levels. The utilization of this assay enables the precise detection of n-oxPTH levels and facilitates the evaluation of their correlation with poor prognosis in patients with CKD. However, the current available clinical research findings indicate that n-oxPTH does not demonstrate clinical superiority over iPTH. Here, we provide a comprehensive review on the mechanism of PTH oxidation, the n-oxPTH assay method, and its correlation with iPTH and clinical outcomes.

## Introduction

Chronic kidney disease-mineral and bone disorder (CKD-MBD) is a common complication of CKD that involves dysregulated mineral and bone metabolism, abnormalities in bone structure, and vascular calcification. Parathyroid hormone (PTH), an 84-amino acid single-chain peptide, is synthesized by the parathyroid gland and plays a major role in the regulation of serum calcium and phosphate levels (1). Besides, PTH also serves as a significant risk factor for increased incidence of cardiovascular disease and overall mortality among CKD patients (2, 3). Therefore, the precise detection of PTH holds immense clinical significance.

Currently, the commonly employed methods for PTH detection in clinical practice are second- and third-generation immunoassays. The second-generation assays, referred to as iPTH assays, possess the capability to capture both full-length PTH and C-terminal PTH fragments present in the blood. On the contrary, the third-generation assay only detects the full-length PTH molecule, referred to as whole PTH assay or bioactive PTH assay.

The PTH hormone possesses two methionines, at position 8 and 18, which are susceptible to oxidation (4). This oxidative process generates three distinct oxidized PTH (oxPTH): ox (Met8) PTH, ox (Met18) PTH, and ox(Met8, 18) PTH (5). Studies have demonstrated that oxidation of PTH leading to its molecular structure change and influence its binding affinity to receptors, especially for ox (Met8) PTH (4, 6, 7). However, PTH oxidation has been neglected in the detection process for several decades. Recently, some researchers have developed a novel technique for determining non-oxidized PTH (n-oxPTH) by selectively removing oxPTH. With this emerging detection technology comes significant interest whether detection of n-oxPTH add more value in diagnosing and treating CKD. A review published in 2020 (8) examined the effects of oxidation on the biological activity of PTH, the mechanisms of PTH oxidation, and the clinical significance of n-oxPTH. However, several new articles have been published since that time, providing additional information regarding PTH oxidation and its measurement. We conducted a comprehensive review of the literature, emphasizing novel detection methods and the relationship between n-oxPTH and clinical outcomes in patients with CKD. In this review, we provided a comprehensive research progress on oxidation of PTH, encompassing the physiological role of PTH, the mechanism underlying PTH oxidation, the detection methodology for n-oxPTH, the relationship between n-oxPTH and iPTH, and the association between n-oxPTH and outcomes in patients with CKD.

## Physiology and metabolism of PTH

PTH is a polypeptide consisting of 84 amino acids that is secreted by the chief cell of the parathyroid gland. The initial synthesis of PTH involves the production of pre-pro-PTH, a 115-amino acid polypeptide that undergoes further cleavage to generate pro-PTH consisting of 90 amino acids. Subsequently, this substance also undergoes intracellular cleavage and becomes an 84-amino acid polypeptide known as PTH, the biologically active form of the hormone (9). It has a molecular weight of approximately 9500 Dalton.

Hypocalcemia (10, 11), hyperphosphatemia (12), and decreased serum 1,25-dihydroxyvitamin D (1,25(OH)_2_D) (13, 14) can stimulate the secretion of PTH, while elevated serum calcium (15), 1,25(OH)_2_D or fibroblast growth factor 23 (FGF23) (16) can inhibit PTH secretion. The rapid secretion of PTH is primarily determined by the extracellular concentration of ionized calcium. After secretion, PTH is taken up by liver and kidney cells where it undergoes intracellular proteolysis to generate active amino-terminal (N-terminal) fragments and inactive carboxy-terminal (C-terminal) fragments. These two types of PTH fragments have distinct fates: the N-terminal fragment is rapidly degraded within liver and kidney cells, whereas the C-terminal fragment predominantly enters circulation and finally excreted by the kidney (17). The half-life of PTH is relatively short, lasting only about 2-5 minutes (18, 19).

PTH plays a crucial role in maintaining the homeostasis of calcium and phosphate levels, with its primary effects mediated through the activation of type 1 PTH receptors (PTH1R), which belong to the G protein-coupled receptor family (20, 21). The main effects of PTH on bone include promoting bone resorption and remodeling, mobilizing calcium from the bone into circulation, facilitating the release of calcium and phosphorus into the extracellular fluid, ultimately leading to an increase in serum calcium concentration (22, 23). In terms of renal effects, PTH promotes calcium reabsorption in renal tubules thereby enhances blood calcium levels (24, 25). Additionally, it reduces phosphate reabsorption in the renal tubules, leading to lower blood phosphorus levels (26). Furthermore, it increases the activity of renal tubular 1-α hydroxylase and promotes the synthesis of (1,25(OH)_2_D) (27, 28).

## Mechanism of PTH oxidation

**Figure 1:**
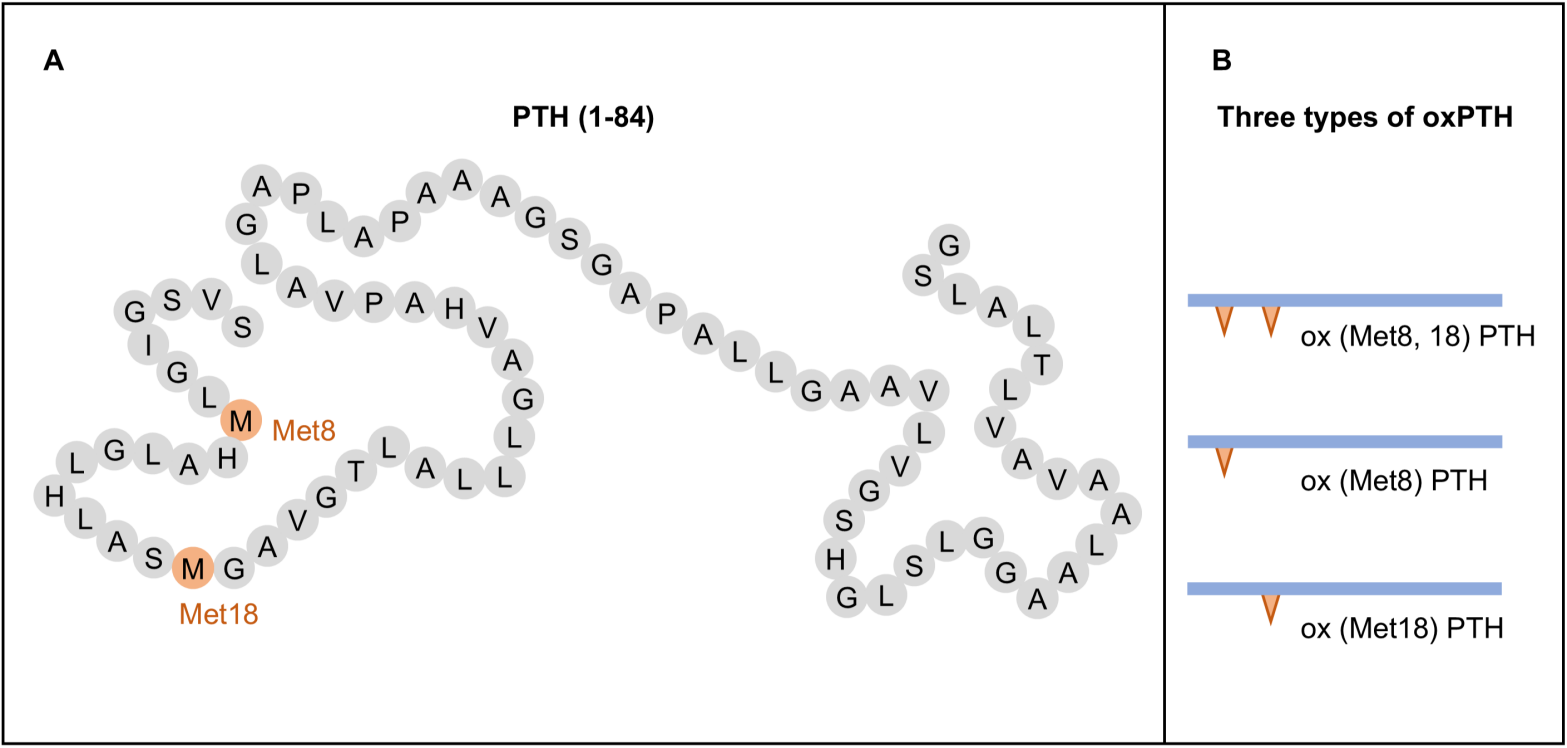
Structure and oxidation products of parathyroid hormone (PTH). **(A)** Structure of PTH (1-84). **(B)** Types of oxidized PTH (oxPTH).

Methionine is easily to be oxidized and form methionine sulfoxide (29). The PTH molecules can undergo oxidation at the methionine residues 8 and 18 (**Figure 1A**), resulting in the generation of three distinct types of oxPTH: ox (Met8) PTH, ox (Met18) PTH, and ox(Met8, 18) PTH (8, 30, 31) (**Figure 1B**). The oxidation of PTH is frequently observed in patients with CKD or patients with maintenance hemodialysis, as these individuals are exposed to increased levels of oxidative stress.

This oxidation process was found to induce structural modification, leading to subsequent alterations in the three-dimensional conformation of PTH (32, 33). Consequently, oxidation significantly impacts the interaction between PTH and its receptor (32). Biological activity of PTH could be assessed by quantifying the enzymatic activity of adenylyl cyclase, which catalyzes the conversion of adenosine triphosphate (ATP) to cyclic adenosine monophosphate (cAMP). Adenylyl cyclase plays a crucial role in PTH signal transduction, leading to an elevation in intracellular cAMP levels upon PTH stimulation (34). Numerous studies have demonstrated that oxPTH fails to induce cAMP production and consequently exhibits inadequate receptor activation compared to n-oxPTH (6, 35–42). This phenomenon can largely be attributed to the oxidation of Met8, rather than Met18 (6). Also, infusion of oxPTH *in vivo* did not induce any changes in serum calcium and urine phosphate concentrations, unlike unmodified PTH (4, 43). Daley et al. (6) reported a noteworthy finding that oxidation of Met8 in the long-acting analog of PTH caused little to no change in its functional interaction with the PTH receptor. This finding demonstrated that oxidation of Met8 in structurally distinct PTH ligands can have markedly different effects.

**Figure 2:**
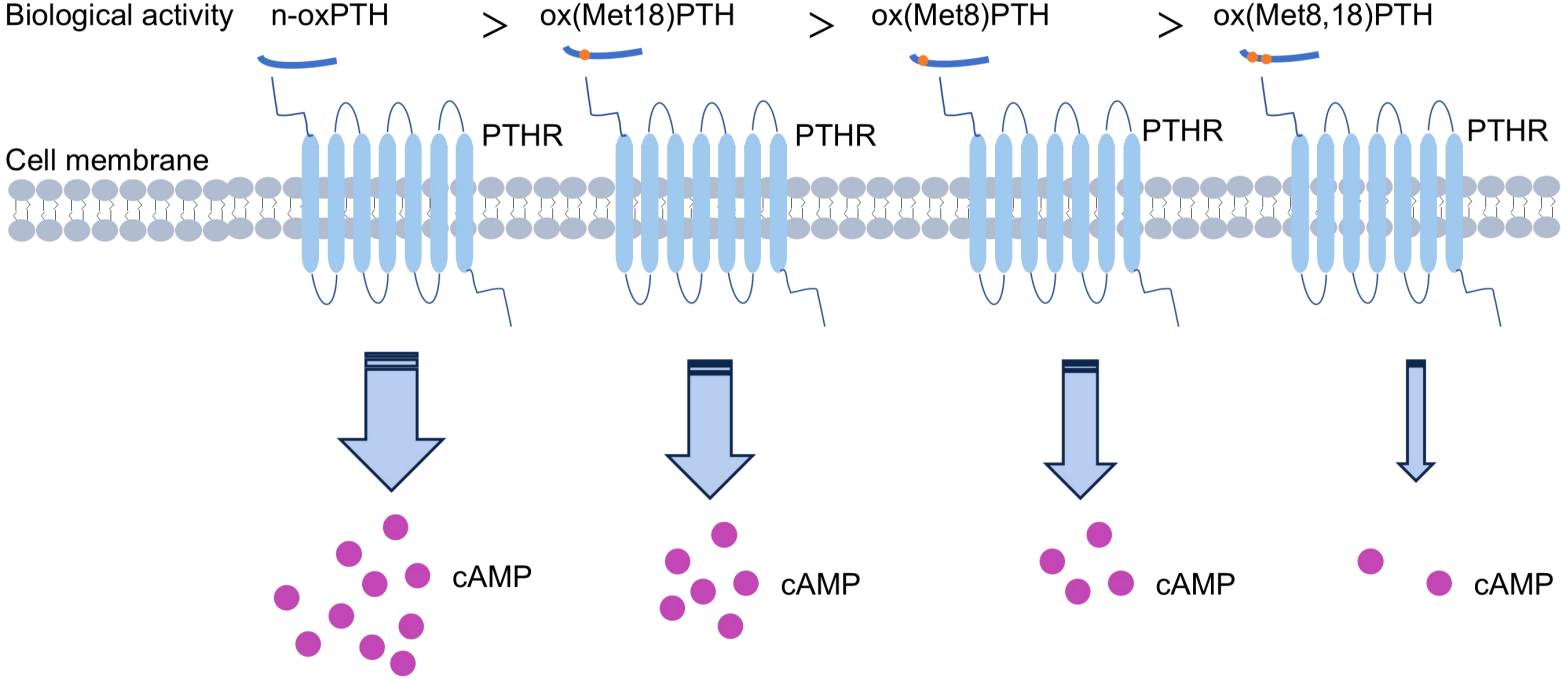
Biological activities of different forms of oxidized parathyroid hormone (oxPTH).

Additionally, various methodologies were employed by researchers to evaluate the biological activity of PTH, encompassing the assessment of renal and bone adenylate cyclase activity, renal excretion of calcium and phosphate, as well as mitochondrial ATPase activity. The findings demonstrated a hierarchical order in terms of PTH’s biological activity: n-oxPTH > ox (Met18) PTH > ox (Met8) PTH > ox(Met8, 18) PTH (7, 31, 44) (**Figure 2**). Furthermore, oxidation occurs more readily in ox(Met18)PTH compared to ox(Met8)PTH (7). The oxidation of Met(8) significantly reduces PTH binding to its receptor, while the oxidation of Met(18) has a limited influence on receptor binding (32, 45, 46). Notably, when both residues are oxidized, PTH exhibits the weakest affinity for the receptor. This result was aligning with observed biological activities.

## Assay of non-oxidized PTH

First-generation PTH measurement kits employ an antibody targeting either the C-terminal or mid-terminal region of PTH (47), resulting in measurement of numerous biologically inactive C-terminal fragments; hence, this assay is no longer employed in clinical nowadays.

Second-generation assay utilizes two types of antibodies: one specifically targeting the N-terminal region of PTH (amino acids 1-34) and another designed to bind to the C-terminal of PTH (amino acids 39-84) (48). Consequently, this assay was designed to exclusively measure intact PTH molecules known as iPTH assay. This assay exhibits a substantial enhancement in both specificity and sensitivity. The iPTH results demonstrated greater consistency with the clinical presentation of patients; however, in certain patient populations such as those with severe secondary hyperparathyroidism, iPTH values were still overestimated (49–51). Due to the absence of N-terminal antibody targeting the first four amino acids, this method has been found to also detect C-terminal fragments, primarily the PTH fragment 7-84 (52).

The third-generation of PTH testing is referred to as whole PTH assay or bioactive PTH assay. This method incorporates an antibody targets C-terminal PTH, along with an antibody specifically targets the initial four amino acids of PTH (53). Consequently, this approach exclusively quantifies full-length PTH in circulation.

Both second- and third-generation PTH immunoassays represented a significant advancement toward the objective of measuring bioactive PTH. Though these methods are widely utilized in clinical settings, there are still some limitations of these assays. On one hand, the PTH testing lacks internationally recognized standards for traceability. Manufacturers use their own calibration standards leading to incomparable results, particularly for CKD patients (54–57). It is worth noting that new international traceability standards for PTH have already been established (IS 95/646), and they will be gradually implemented in the future for clinical detection of PTH. On the other hand, currently used PTH assays overlook the crucial post-translational modification process involving PTH oxidation.

**Figure 3:**
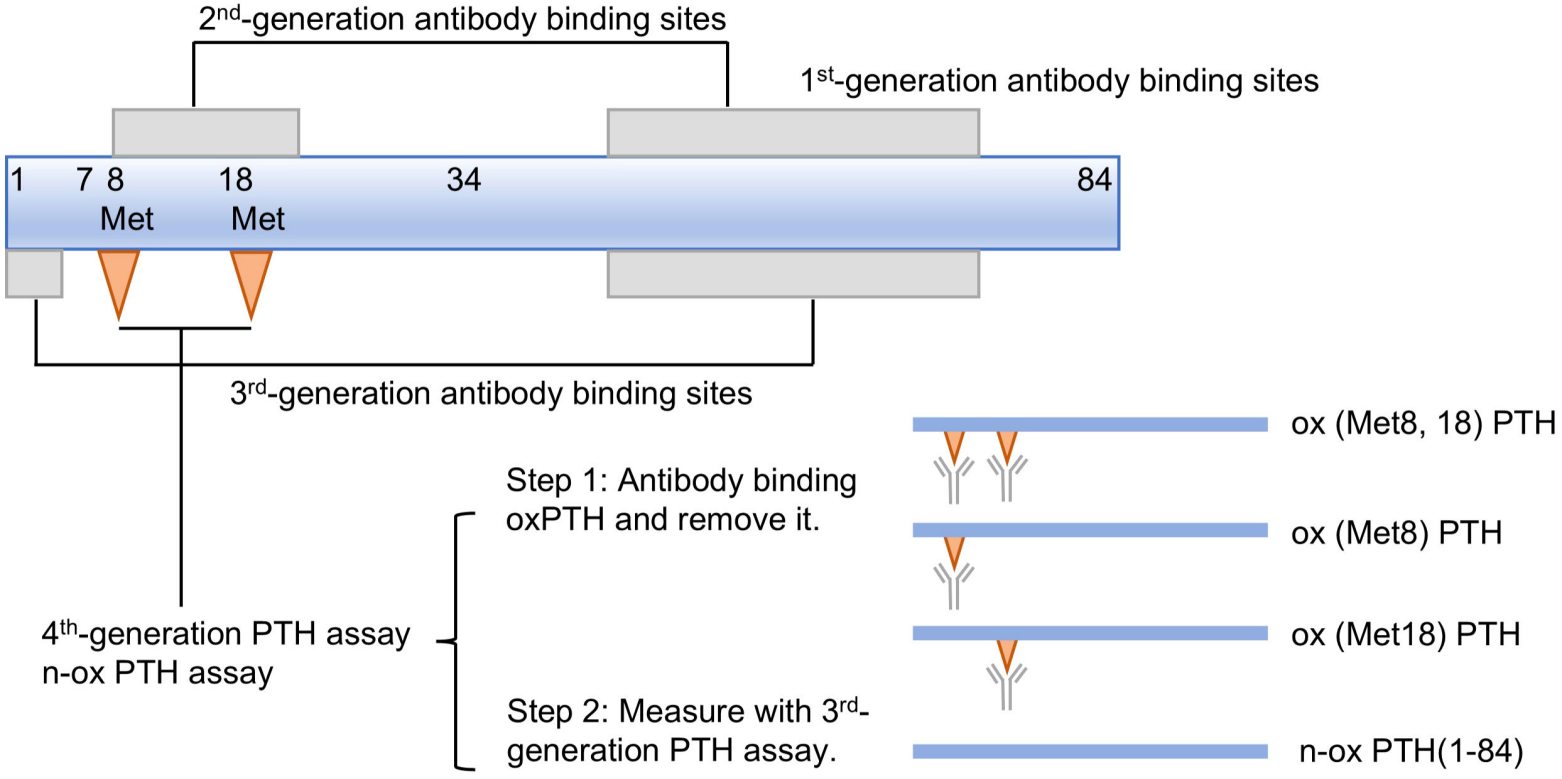
First to fourth-generation parathyroid hormone (PTH) assays.

Previous studies have revealed that the oxidation of PTH can significantly impact its biological activity, hence, it is imperative to detect n-oxPTH for accurate assessment of the real PTH level. To address this limitation, Hocher et al. (30) established a method for determining n-oxPTH recently. This novel test process comprises of two sequential steps. Initially, a specific affinity chromatography column is employed to eliminate any oxPTH including ox (Met8) PTH and/or ox (Met18) PTH from the sample using antibody targeting oxPTH. Subsequently, the remaining n-oxPTH is subjected to analysis by third-generation PTH assay system (**Figure 3**). It is worth noting that the n-oxPTH detection method eliminates all forms of oxPTH and does not differentiate between ox (Met8) PTH, ox (Met18) PTH, and ox (Met8, 18) PTH, which may have different biological activities. Liquid chromatographs coupled with tandem mass spectrometers (LC-MS/MS) have recently been employed for the detection of PTH (58, 59). This detection offers the advantage of exceptional sensitivity; however, it is accompanied by the drawback of complex sample preparation. Specifically, tryptic digestion and subsequent fragment quantification are required for large peptides like PTH. This process may lead to the loss of additional information, such as oxidation or newly formed fragments. Ideally, a selective and gentle technique is needed to differentiate PTH and its derivatives in their native state.

## The association between n-ox PTH and iPTH

With the development of the n-oxPTH assay by Hocher et al., the detection of bioactive non-oxidizing PTH levels *in vivo* has been facilitated, leading to an increased interest in the clinical significance of n-oxPTH measurement. In the initial clinical research article conducted by Hocher’s team (30), they examined the levels of n-oxPTH and iPTH in 18 dialysis patients and demonstrated that only 7-34% traditionally measured PTH were real intact PTH. Subsequently, they evaluated n-oxPTH concentrations in a larger cohort (32) consisting of 620 children with CKD stages 2-4, 342 adult patients on dialysis, 602 kidney transplant recipients, and 89 healthy controls. The results indicated a significant correlation between n-oxPTH and age, while no significant association was found with sex. The level of n-oxPTH were 1.5-2.25 times increased in patients with renal failure compared with controls. Zuo et al. (60) assessed the relationship of n-oxPTH and iPTH in 531 stable kidney transplant recipients and found that the association between iPTH and oxPTH was stronger than the association between iPTH and n-oxPTH. The findings of this study indicate that over 85% of iPTH underwent oxidation. Cavalier et al. (61) examined the levels of iPTH and n-oxPTH in 66 stable hemodialysis patients at baseline and after 1, 3, 6, and 12 months. The findings demonstrated that the n-oxPTH fraction consistently accounted for 11.3% (9.0-13.7%) of iPTH throughout the study duration, indicating a stable proportion of n-oxPTH in stable hemodialysis patients. All of these results suggest that the measurement of n-oxPTH may provide a more accurate reflection of the hormonal status, as opposed to iPTH which solely indicates oxidative stress in patients with renal failure.

However, contrary findings have been reported in some studies. Kritmetapak et al. (62) developed a liquid chromatography-high resolution mass spectrometry (LC-HRMS) method for the measurement of PTH in 221 patients with stages 1-5 CKD. Their results showed that LC-HRMS could identify the existance of PTH1-84, C-terminal PTH fragments, and mid-region PTH fragments in blood of patients with CKD. The levels of both PTH1-84 and PTH fragments were elevated in patients with decreased renal function. Furthermore, it was noted that concentrations of PTH measured using LC-HRMS tended to be lower than those obtained through iPTH detection. Importantly, the results from this study did not support the presence of PTH7-84 or oxPTH in blood. Thus, whether n-oxPTH should be detected in clinical diagnosis and treatment is still controversial.

## The association between n-oxPTH and clinical outcomes

Zeng et al. (63) evaluated the relationship between n-oxPTH levels and FGF23 concentrations in two distinct cohorts of patients, including 620 children with CKD (eGFR 10-60 ml/min/1.73m^2^) and 600 kidney transplant patients. Their findings revealed a significant association between n-oxPTH and FGF23 concentrations, while no such relationship was observed for oxPTH. Furthermore, as kidney function declined progressively, both iPTH and oxPTH exhibited substantial increases, whereas n-oxPTH showed only moderate elevation. This study revealed that n-oxPTH may be related to mineral metabolism in patients with CKD. However, Ursem et al. (64) compared the efficacy of n-oxPTH and iPTH as markers for assessing bone turnover in 31 patients with end stage kidney disease (ESKD) and found that both iPTH and n-oxPTH were strongly correlated with histomorphometric and circulating parameters of bone turnover. Consequently, at present, measuring n-oxPTH does not offer any additional value compared with iPTH when evaluating bone turnover in patients with renal failure.

The correlation between n-oxPTH and prognosis has been examined in several clinical studies, as summarized in **Table 1**. However, the predictive value of n-oxPTH for poor prognosis in CKD patients remains a subject of controversy. Lu et al. (65) detected circulating PTH concentrations in 600 kidney transplant patients. Then these patients were followed-up for graft loss for 3 years. Their results demonstrated a significant association between graft loss and n-oxPTH, while no significant associations were observed with oxPTH or iPTH. In another study, Seiler-Mussler et al. (66) measured PTH levels in 535 patients with CKD (eGFR 10-60 ml/min/1.73m^2^) and followed them for five years to assess the risk of poor prognosis. The findings demonstrated that there was no significant association between n-oxPTH and clinical outcomes in non-dialysis CKD patients, including atherosclerotic events, acute heart failure, CKD progression, and all-cause mortality. The correlation between n-oxPTH and risk of death in 340 hemodialysis patients was analyzed over a 5-year follow-up period (67). The results revealed that patients with elevated n-oxPTH levels exhibited a higher survival rate in comparison to the group with lower n-oxPTH levels. However, the mortality in a subset of patients with iPTH concentrations exceeding the upper limit of the iPTH assay (70 ng/L) was found to be associated with oxPTH concentrations, rather than n-oxPTH concentrations. Hence, the controversy persists regarding whether measures of iPTH primarily reflect the oxidative stress status in CKD patients rather than their PTH hormone levels. The potential of n-oxPTH detection to enhance clinical diagnosis, treatment, and prognosis analysis necessitates further exploration by more RCT studies.

**Table 1:** Clinical studies evaluated non-oxidized PTH.

Reference	Subjects	PTH assay	Conclusions
Zuo et al., 2023 (60)	531 kidney transplant patients	iPTH measured by Roche; n-oxPTH measured after oxPTH was removed by anti-human oxPTH monoclonal antibodies	OxPTH had a strong relationship with iPTH (r=0.998, *P*<0.0001), n-oxPTH was not that correlated with iPTH (r=0.866, *P*<0.0001).
Kritmetapak et al., 2021 (62)	221 patients with stage 1-5 CKD	PTH1-84 and PTH fragments were measured using LC-HRMS. PTH was also measured using iPTH immunoassay (Roche).	Serum PTH1-84 and its fragments increase as eGFR declines. Serum PTH concentrations measured using LC-HRMS were lower than iPTH immunoassay. PTH7-84 and oxidized forms of PTH1-84 were not detected.
Ursem et al., 2021 (64)	31 patients with ESKD who underwent bone biopsy	iPTH measured by Roche; n-oxPTH measured after oxPTH was removed by anti-human oxPTH monoclonal antibodies	The n-oxPTH values were 12% of iPTH. N-oxPTH is not superior to iPTH as a biomarker of bone turn over.
Zeng et al., 2020 (63)	620 children with CKD (eGFR 10-60 ml/min/1.73m^2^) and 600 kidney transplant recipients	iPTH measured by Roche; n-oxPTH measured after oxPTH was removed by anti-human oxPTH monoclonal antibodies	N-oxPTH, but not oxPTH, was significantly associated with FGF23 concentrations. The increase in PTH with decreasing GFR is mainly due to an increase in oxPTH.
Lu et al., 2020 (65)	600 kidney transplant recipients.	iPTH measured by Roche; n-oxPTH measured after oxPTH was removed by anti-human oxPTH monoclonal antibodies	OxPTH had a strong correlation with iPTH, whereas the correlation between n-oxPTH and iPTH was weaker. Only n-oxPTH but not oxPTH nor iPTH is associated with graft loss.
Cavalier et al., 2020 (61)	66 ESKD patients undergoing hemodialysis	iPTH measured by Roche; n-oxPTH measured after oxPTH was removed by anti-human oxPTH monoclonal antibodies	The ratio n-oxPTH/iPTH ranged from 5.1 to 35.7%. The percentage of n-ox PTH is stable over time in stable HD patients.
Ursem et al., 2019 (68)	108 vitamin D-insufficient (25(OH)D <75 nmol/L) hypertensive patients with a mean eGFR of 83 ml/min/1.73m^2^)	iPTH measured by Roche; n-oxPTH measured after oxPTH was removed by anti-human oxPTH monoclonal antibodies	Upon vitamin D treatment, both iPTH and n-oxPTH concentrations decreased leading to a significantly increased ratio of n-oxPTH/iPTH.
Seiler-Mussler et al., 2018 (66)	535 patients with CKD (GFR 15-89ml/min per 1.73 m^2^).	iPTH measured by Roche; n-oxPTH measured after oxPTH was removed by anti-human oxPTH monoclonal antibodies	In patients with CKD, iPTH was associated with all-cause mortality; there was no association of n-oxPTH with any of the clinical outcomes examined.
Ursem et al., 2017 (69)	17 ESKD patients undergoing hemodialysis and 32 healthy subjects	iPTH measured by Roche; n-oxPTH measured after oxPTH was removed by anti-human oxPTH monoclonal antibodies	N-oxPTH concentrations were stable indicating negligible ex vivo oxidation of PTH.
Tepel et al., 2013 (67)	340 ESKD patients undergoing hemodialysis	wPTH measured by Roche; n-oxPTH measured after oxPTH was removed by anti-human oxPTH monoclonal antibodies	The predictive value of n-oxPTH and wPTH on the mortality of hemodialysis patients differs substantially. Measurements of n-oxPTH may reflect the hormone status more precisely.
Hocher et al., 2013 (32)	620 children with CKD stage 2-4, 342 adult ESKD patients on dialysis, and 602 kidney transplant recipients	iPTH measured by Roche; n-oxPTH measured after oxPTH was removed by anti-human oxPTH monoclonal antibodies	A huge proportion of iPTH is oxPTH. The concentrations of n-oxPTH were 1.5-2.25 times higher in patients with CKD compared with controls. Measurements of n-oxPTH may reflect the hormone status more precise.
Hocher et al., 2012 (30)	18 ESKD patients on dialysis	iPTH measured by Roche; n-oxPTH measured after oxPTH was removed by anti-human oxPTH monoclonal antibodies	A huge but not constant proportion of PTH molecules are oxidized in patients requiring dialysis.

PTH, parathyroid hormone; iPTH, intact PTH; oxPTH, oxidized PTH; n-oxPTH, non-oxidized PTH; CKD, chronic kidney disease; LC-HRMS, liquid chromatography-high resolution mass spectrometry; ESKD, end stage kidney disease; FGF23, fibroblast growth factor 23; GFR, glomerular filtration rate; wPTH, whole PTH.

Currently, apart from the utilization of human n-oxPTH monoclonal antibody for the elimination of oxPTH from blood, there is no superior approach available for quantifying n-oxPTH levels. Furthermore, different instruments yield disparate quantitative outcomes for PTH levels. Existing PTH immunoassays also employ inconsistent standards. It should be noted that the widespread implementation of n-oxPTH measurements has been impeded due to the aforementioned limits. Measuring n-oxPTH levels may provide a more accurate and real PTH status in patients with CKD, nevertheless, current evidence does not support the clinical value of measuring n-oxPTH.

## Conclusions

PTH is an important biomarker need to be monitored regularly in patients with CKD. At present, detection of PTH primarily relies on second- or third-generation immunoassays. The oxidation of PTH *in vivo* can result in a loss of its biological activity. Recently, researchers have made significant advancements in the detection of n-oxPTH in blood. It is important to note that this assay does not differentiate different oxidation forms of PTH, whereas ox (Met8) PTH, ox (Met18) PTH, and ox (Met8,18) PTH exhibit different biological activities. Therefore, further optimization of bioactive PTH measurement methods is still required to determine if current techniques excessively exclude all forms of oxPTH.

Many clinical studies have revealed the correlation between all-cause mortality, cardiovascular events and iPTH levels. Furthermore, it has been reported that iPTH exhibits a robust correlation with oxPTH and a relatively weak correlation with n-oxPTH. Therefore, accurate assessment of n-oxPTH may hold greater value in determining the correlation between PTH and patient prognosis. Several clinical studies analyzed the correlation between n-oxPTH and outcomes, results showed that measuring n-oxPTH does not yield any additional clinical value. The suboptimal outcome may be attributed to the fact that current n-oxPTH tests fail to differentiate between different forms of oxPTH. It is imperative to develop novel PTH detection methods capable of discerning different oxidation states, including n-oxPTH, ox (Met8) PTH, ox (Met18) PTH, and ox (Met8, 18) PTH. Such an innovative test has the potential to bring more clinical significance. Until a more refined assay is devised, it is advisable to continue employing second- or third-generation PTH tests currently utilized in clinical practice.
